# A prospective investigation of depression and adverse outcomes in patients undergoing vascular surgical interventions: A retrospective cohort study using a large mental health database in South London

**DOI:** 10.1192/j.eurpsy.2021.2

**Published:** 2021-01-18

**Authors:** Sajini Kuruppu, Marvey Ghani, Megan Pritchard, Matthew Harris, Ruwan Weerakkody, Robert Stewart, Gayan Perera

**Affiliations:** 1 Department of Psychological Medicine, Institute of Psychiatry, Psychology & Neuroscience. King’s College London. United Kingdom; 2 SLaM BRC Nucleus, South London and Maudsley NHS Foundation Trust. London. United Kingdom; 3 Department of Vascular Surgery, The Royal Free Hospital, Pond Street, London NW3 2QG, United Kingdom; 4 King’s College Hospital NHS Foundation Trust, Denmark Hill, London, United Kingdom

**Keywords:** Admission, cardiovascular, depression, mortality, surgery

## Abstract

**Background:**

Patients with depression are more susceptible to cardiovascular illness including vascular surgeries. However, health outcomes after vascular surgery among patients with depression is unknown. This study aimed to investigate associations of depression with post-operative health outcomes for vascular surgical patients.

**Methods:**

A retrospective observational study was conducted using data from a large mental healthcare provider and linked national hospitalization data for the same south London geographic catchment. OPCS-4 codes were used to identify vascular procedures. Health outcomes were compared between those with/without depression including length of hospital stay (LOS), inpatient mortality, and 30 day emergency hospital readmissions. Predictors of these health outcomes were also assessed.

**Results:**

Vascular surgery was received by 9,267 patients, including 446 diagnosed with depression. Patients with depression had a higher risk of emergency admission for vascular surgery (odds ratio [OR] 1.28; 1.03, 1.59), longer index LOS (IRR 1.38; 1.33–1.42), and a higher risk of 30-day emergency readmission (OR 1.82; 1.35–2.47). Patients with depression had higher inpatient mortality after adjustment for sociodemographic status (1.51; 1.03, 2.23) but not on full adjustment, and had longer emergency readmission LOS (1.13; 1.04, 1.22) after adjustment for sociodemographic factors and cardiovascular disease. Correlates of vascular surgery hospitalization among patients with depression included admission through emergency route for longer LOS, inpatient mortality, and 30-day hospital readmission.

**Conclusion:**

Patients with depression undergoing vascular surgery have substantially poorer health outcomes. Screening for depression prior to surgery might be indicated to target preventative measures.

## Introduction

Depression has a high prevalence among patients with cardiovascular disease (CVD), including coronary heart disease and peripheral arterial disease (PAD) [1, 2]. This association is in part thought to be mediated by the hyperactivity of the immune system’s inflammatory response, whereby chronic inflammation is associated with a greater risk of future cardiac events and mortality [3, 4]. PAD is independently associated with increased pro-inflammatory markers in people with depression, and is in itself associated with higher mortality risks [5–7]. Considering the potential need for vascular surgery in PAD, individuals with depression have been found to have greater post-operative pain for surgical procedures in general, which in turn has been associated with decreased patient satisfaction and increased pulmonary and cardiac complications, increased mortality and morbidity, and a greater likelihood of developing chronic pain [8–11]. Furthermore, inadequate self-health monitoring has been suggested in people with depression compared to those without mental disorders. For example, it is reported that individuals with depression may only decide to seek medical advice when their illness has progressed significantly, and when they do, they may be less likely to follow the recommendations offered to them by clinicians [12, 13]. This poor communication between clinicians and patients may also result in treatment being delayed even further [12]. Additionally, people with depression may adopt unhealthier habits such as smoking or excessively drinking alcohol; habits that are potentially related to their mental health difficulties [14].

Overall, the combination of predisposing mechanisms and inequalities in healthcare receipt increases the risk of patients with depression having poorer health outcomes following vascular surgery. For example, PAD patients with depression have been described as being at increased risk of severe progression of PAD symptoms, increased mortality, increased re-intervention, and failure rates following peripheral revascularization, and a greater likelihood of requiring major amputations [15–18]. Overall, higher mortality rates have been observed following cardiac or vascular surgeries, for patients with serious mental illnesses with affective symptoms, such as bipolar disorder, post-traumatic stress disorder [19, 20] and major depressive disorder, suggesting that depressive mood episodes are particularly associated with this risk. Additionally, psychiatric comorbidities such as anxiety have been associated with greater post-operative mortality and morbidity, post-surgical cardiac complications, and an increased risk of hospital admissions [21, 22]. Whilst evidence an increased mortality risk has been described in patients with depressive symptoms following cardiovascular surgery, post-operative health outcomes more generally have received relatively little investigation. Furthermore, previous participant samples have had relatively limited generalizability. Other factors found to be associated with poorer surgical outcomes, and therefore important to account for in investigations, have included cessation of psychotropic medication prior to surgery [23], lower household income [24], hypertension, older age, female gender, African American ethnicity [25], and substance abuse such as cannabis use [26, 27] and opioid use [28]. Furthermore, there is a need to take into account the different vascular surgical procedures most commonly received and/or recommended in healthcare guidelines [29].

Therefore, this study aimed to quantify standardized admission ratios (SARs) for vascular surgery in patients with diagnosed depression, and to compare a broad range of post-operative health outcomes between patients with depression and general population counterparts. A secondary aim was to investigate predictors of health outcomes following vascular surgery including four surgery categories to compare findings for such patients: Aortic/visceral, endovascular, Major vascular, Peripheral endovascular and Other, in accordance with The National Institute for Health and Care Excellence (NICE) guidelines [29].

## Methods

### Study setting and data source

A retrospective observational study was conducted using data from the South London and Maudsley NHS Foundation Trust (SLaM) Biomedical Research Centre Case Register which extracts data from a mental healthcare provider (SLaM) serving a defined geographic catchment of around 1.3 million residents in Lambeth, Lewisham, Southwark, and Croydon boroughs of south London [30]. The Clinical Record Interactive Search (CRIS) application was developed in 2007–2008 with National Institute for Health Research funding to provide de-identified copies of SLaM’s electronic health record [31] for research purposes, and has been subsequently enhanced through natural language processing applications using Generalized Architecture for Text Engineering software, extracting information from text fields [30]. Data are currently archived in CRIS on over 500,000 cases with a range of mental disorders, CRIS has been linked with data from a variety of sources including national Hospital Episode Statistics (HES), and the database (including linkages) has full approval for secondary analysis (Oxford Research Ethics Committee C, reference 18/SC/0372). The study described here was covered by this database approval.

### Sample

Using the linkage between CRIS and HES, patients were ascertained who had vascular surgical procedure and who were living in SLaM’s catchment between 1st January 2007 and 31st March 2018. Vascular surgery procedures were identified from HES using the OPCS-4 codes outlined in the Supplementary Table S1. Using these data two cohorts were created.

Initially, all patients who had vascular surgery procedure residing in SLaM catchment boroughs were identified (*n* = 10,824). The few patients who had missing data related to age and gender were excluded (*n* = 86), followed by exclusion of patients who were aged under 18 at the time of vascular surgery (*n* = 336). This left 10,402 adults who had received vascular surgery in this cohort. Out of this number, 1,581 patients had received mental health assessment or care from SLaM prior to the first instance of vascular surgery. Cohort 1 comprised patients who had received a diagnosis of depression (defined as ICD-10 codes F32 or F33; *n* = 446), and Cohort 2 comprised the remainder of adults living in the SLaM catchment area who had no contact with SLaM (*n* = 8821).

Once the cohorts were assembled, the types of vascular surgery were classified into four groups, in accordance with NICE guidelines [29]: “Aortic/visceral Endovascular,” “Major open vascular,” “Peripheral Endovascular,” and “Other.” This was to reflect differences in morbidity anticipated to be associated with different vascular procedures and to allow appropriate comparison of post-operative health outcomes.

### Measurements

The index date for defining exposure and confounding variables was the first date of admission to hospital for vascular surgery during the surveillance period. At the index date, demographic status was ascertained for patients, including age at vascular procedure, gender, ethnicity (White and non-White), marital status (cohabiting or non-cohabiting), and index of multiple deprivation (IMD 2015) statistics which include measures of income, employment, education, skills and training, health and disability, crime, barriers to housing and services and living environment for each patient’s neighborhood (UK Lower Super Output Area with population between 1,500 and 2,000) at the time of index date.

Furthermore, Health of the Nation Outcome Scales (HoNOS) item scores and dates were extracted in the year preceding the index date, and the most recent scores were included for analyses. HoNOS are routinely administered in UK mental health services to measure clinical and functional status, and include items related to agitation self-injury, substance abuse, cognitive function, physical health, hallucinations, depressed, relationship, daily living, living conditions, and occupational status, with severity measured on 5-point Likert scales. For our analysis score of 0 and 1 is defined as no problem and score 2, 3, 4 is defined as having a problem.

Other recorded psychiatric comorbidities, derived from structured diagnosis, were also measured in the year preceding the index date including: (a) mental and behavioral disorders due to psychoactive substance use (ICD-10 diagnosis F10–F19); (b) dementia (F00, F01, F02, F03); (c) neurotic, stress-related, and somatoform disorders (F40–F48); (d) eating disorders (F50); (e) disorders of adult personality and behavior (F60–F69).

Medications taken by participants 1 year before the index date were identified in CRIS, extracted through natural language processing, and use of the following were ascertained: antipsychotics, antidepressants, antihypertensives (any), hypnotics, alpha blockers, antihypertensives, analgesics, anxiolytics and hypnotics, beta blockers, calcium channel blockers, diuretics, angiotensin II receptor antagonists (Sartans), antidiabetes medication, and anticoagulants. Subclasses of antihypertensives were not extracted in this study.

Hospital admission information was obtained within the year prior to the index date and CVD related admissions were identified through ICD-10 diagnosis data: (a) ischemia and coronary heart disease (I20–I25); (b) arrhythmia (I44–I49); (c) heart failure (I50); (d) diabetes (E08–E13); (e) hypotension (I95–I99); (f) hypercholesterolemia (E78); (g) hypertension (I10–I15). The following physical disability-related hospital admissions were ascertained based on first listed discharge diagnosis: (a) urinary tract infections (UTIs) (N39); (b) osteoporosis (M80–M85); (c) visual disturbance; (d) syncope or collapse (R50–R69).

Emergency hospital admissions were identified using “admission methods” codes 21–24 and 28 as per NHS data dictionary definition [32]. Elective admissions were defined from codes 11–13.

The following health outcomes were measured during the index vascular surgery hospital spell:

1. Type of hospital admission for vascular surgery (emergency or elective; odds ratio [OR]).
2. Length of hospital stay (LOS) for vascular surgery from date of admission to date of discharge (incidence rate ratio (IRR) from Poisson regression models).
3. Patient mortality following vascular surgery, as identified through HES (OR).

The following outcomes were measured after the index admission in people who were alive at discharge:

1. Readmission to hospital as an emergency within 30 days following discharge (OR).
2. LOS for emergency readmission as outlined in outcome 4, from the date of admission to date of discharge (IRR from zero-inflated Poisson regression models).

### Statistical analysis

Vascular surgery SARs adjusted for age and gender were ascertained for patients with depression (Cohort 1) who were admitted to hospital for vascular surgery either via an elective or emergency route and compared against all adults who had received vascular surgery. When calculating SARs, all adults who had vascular surgery, consisting of the SLaM catchment population (Lambeth, Southwark, Lewisham, and Croydon residents) was used as the standard reference population. SAR for vascular surgery was calculated between 2007 and 2018 covering an average of 98,106 annual adults (age 18 and over) residing in the SLaM catchment area (ONS population estimations). Out of this initial catchment population, 374,332 residents were in contact with SLaM services and 67,558 adults were diagnosed with depression. Out of this sample of patients with depression, those who underwent vascular surgery were ascertained and included in SAR calculation in comparison to those all adults who had vascular surgery in the SLaM catchment population. Indirect age and gender standardization method was used to measure SAR by ascertaining age/gender on admission for the catchment population, and generating expected admission rates from the known age/gender structure of the catchment population.

Next, post-operative health outcomes were compared between patients with depression and the general population comparison cohort. Logistic regression analysis and subsequent OR calculations were carried out to investigate associations with admission to hospital via an elective or emergency route, presence or not of inpatient mortality, and whether the patient was readmitted to hospital via emergency route within 30 days of discharge. IRRs from Poisson regression analyses were calculated to investigate factors associated with LOS for the index admission, and zero-inflated Poisson regression analyses were used for LOS for an emergency hospital admission within 30 days. Outcomes were incrementally adjusted for sociodemographic factors, CVD and physical disability-related hospital admissions and final models adjusted for emergency hospital admissions. Next, multivariable analyses were carried out to investigate factors associated with vascular surgery outcomes in patients with depression. Two outcomes were measured: LOS (IRR) and inpatient mortality (OR). Finally, multivariable analyses investigated predictors of health outcomes during emergency readmission to hospital within 30 days after discharge for patients with depression. Two outcomes were investigated: odds of emergency hospital admission within 30 days after index discharge (OR) and LOS for emergency hospital admission within 30 days after index discharge (IRR).

All regression analyses used backward elimination, starting with all candidate variables from unadjusted analysis, testing the deletion of each variable, deleting the variable (if any) whose loss gives the most statistically insignificant deterioration of the model fit (with elimination on the basis of a *p*-value greater than 0.10), and repeating this process until no further variables can be deleted without a statistically significant loss of fit. Akaike’s Information Criteria (AIC) and Bayesian information criteria (BIC) were used to determine the model fit. If AIC/BIC scores were within about 10 of each other, the difference between the two models was considered marginal. Both AIC and BIC were presented in each table. Furthermore, recommended robust standard errors [32] were obtained for the parameter estimates to control for mild violation of underlying assumptions and independent variables which were autocorrelated were eliminated during this process. All statistical analyses were carried out using STATA 13 software.

## Results

Table 1 describes the sociodemographic, psychiatric, physical health, and medical characteristics of the two cohorts. Of the total sample population of 9,267 participants, 95.2% were comprised of the general population in the SLaM catchment area without mental health diagnoses and 4.8% patients had been diagnosed with depression. The cohort of patients with depression had higher proportions of emergency admissions for vascular surgery (46.5%) and fewer elective admissions (53.5%), compared to the comparison cohort of patients in the general population (33.5 and 66.5% for index vascular surgery hospitalizations, respectively). Patients in the comparison cohort were slightly older (62.3 years) compared to patients with depression (59.4 years). Relatively more patients with depression were female (52.6%) as compared with the general population (44.9%). Of the people undergoing vascular surgery, those in the depression cohort were more often white (52.6%) than those from the comparison cohort (44.9%). Area level deprivation score measured by IMD 2015 was not statistically significant between patients with depression and general population. Patients with depression were more likely to have had hospital admissions related to physical disabilities, particularly syncope (34.5% of patients with depression and 6.3% of the general population) and UTIs (19.9% of patients with depression compared to 2.3% of the general population), as well as more hospital admissions related to CVD.

**Table 1.** Characteristics of the study cohort compared with general population.

Characteristics	General comparison population (*n* = 8,821)^**a**^	Patients with depression (*n* = 446)	*p*-Value
Mean (SD) age	62.3 (17.0)	59.4 (17.7)	<0.001^b^
Female gender	3962 (44.9)	235 (52.6)	<0.001^c^
Non-white ethnicity	4361 (49.4)	124 (27.7)	<0.001^c^
Mean neighborhood IMD score (SD)	29.2 (10.9)	29.6 (11.0)	0.45^b^
HoNoS problems			
Agitation problems		35 (7.8)	
Self-injury problems		46 (10.3)	
Substance abuse problems		32 (7.2)	
Cognitive problems		61 (13.7)	
Physical health problems		234 (52.4)	
Hallucinations		21 (4.7)	
Depressed		168 (37.6)	
Relationship problems		87 (19.5)	
Daily living problems		141 (31.5)	
Living conditions problems		54 (12.1)	
Occupational problems		96 (21.5)	
Psychiatric comorbidities before vascular surgery			
Mental and behavioral disorders due to psychoactive substance use (F10–F19)		24 (5.4)	
Dementia		14 (3.1)	
Neurotic, stress-related and somatoform disorders (F40–F48)		29 (6.5)	
Disorders of adult personality and behavior (F60–F69)		21 (4.7)	
Psychiatric medication before vascular surgery			
Antipsychotics		70 (15.7)	
Antidepressants		200 (44.7)	
Anxiolytics and hypnotics		88 (19.7)	
Physical health medication			
Anticoagulants		41 (9.2)	
Antidiabetics		26 (5.8)	
Analgesics		78 (17.5)	
Antihypertensives		88 (19.7)	
Hospital admissions prior to vascular surgery			
*Previous physical disability-related admissions*			
Syncope and collapse	560 (6.3)	154 (34.5)	<0.001^c^
Osteoporosis	135 (1.5)	35 (7.8)	<0.001^c^
UTIs	204 (2.3)	89 (19.9)	<0.001^c^
*Previous CVD hospital admissions*			
Arrhythmia	1060 (12.0)	96 (21.5)	<0.001^c^
Ischemia and coronary heart disease	1571 (17.8)	132 (29.5)	<0.001^c^
Hypertension	4129 (46.8)	270 (60.4)	<0.001^c^
Hypotension	287 (3.3)	61 (13.6)	<0.001^c^
Diabetes	2028 (23.0)	157 (35.1)	<0.001^c^
Heart failure	479 (5.4)	63 (14.1)	<0.001^c^
Hypercholesterolemia	1633 (18.5)	141 (31.5)	<0.001^c^
Type of vascular surgery			
Aortic/visceral endovascular	900 (10.2)	33 (7.4)	0.06^c^
Major open vascular	1362 (15.4)	59 (13.2)	0.21^c^
Peripheral endovascular	2782 (31.5)	146 (32.7)	0.60^c^
Other	3777 (42.8)	208 (46.7)	0.12^c^
Post-operative outcomes			
*Outcomes during vascular surgery*			
*Method of admission for vascular surgery*			
Elective	5868 (66.5)	239 (53.5)	<0.001^c^
Emergency	2953 (33.5)	208 (46.5)	<0.001^c^
Mean (SD) vascular surgery spell length (days)	5.5 (11.6)	11.8 (20.2)	<0.001^b^
Inpatient mortality	421 (4.8)	30 (6.7)	0.06^c^
*Outcomes within 30-days discharge from vascular surgery*			
Had emergency hospital admission	689 (7.8)	73 (16.3)	<0.001^c^
Mean (SD) LOS (days) for emergency hospital readmission	9.4 (17.1)	10.1 (18.8)	0.40^b^

Abbreviations: CVD, cardiovascular disease; IMD, index of multiple deprivation; LOS, length of hospital stay; UTI, urinary tract infection.

a Only included patients without any contacts with South London and Maudsley NHS foundation Trust residing in four catchment boroughs.

b *p*-Value was obtained using *t*-test for mean difference between general comparison population and patients with depression.

c *p*-Value was obtained using chi squared test with one degree of freedom for difference between general comparison population and patients with depression.

In terms of type of surgery, the comparison cohorts were similar with respect to sub-groups (aortic/visceral endovascular, major open vascular, peripheral endovascular, and other) with the highest proportion undergoing peripheral endovascular surgeries, apart from “other” surgeries. Emergency and elective vascular surgery SARs for patients with depression were 3.30 (95% CI: 2.88, 3.78) and 1.79 (95% CI: 1.55, 2.00) respectively, thus showing higher likelihoods of both emergency and elective admissions compared to those expected from the general population.

Table 2 displays analyses of post-operative health outcomes following vascular surgery for the two cohorts, successively adjusted for covariates. In summary, patients with depression were more likely to be admitted to hospital for index vascular surgery via an emergency route than elective route as compared to the general population and had a 38% longer index LOS. Furthermore, patients with depression also had higher odds of emergency readmission to hospital within 30 days of discharge from index hospitalization; however, longer 30-day hospitalizations, were no longer significant when adjusted for physical disability related hospital admissions and emergency vascular hospitalization. Similarly, higher inpatient mortality after adjustment for age, gender, ethnicity, and IMD score, was no longer significant when further adjusted for CVD and physical disability related hospital admissions and emergency vascular hospital admissions.

**Table 2.** Regression analyses of post-operative vascular surgery outcomes for patients with depression compared with those from the general population.

	*Index vascular surgery hospitalization*			*Subsequent readmission*	
Adjustments	OR for emergency route of admission	IRR for LOS	OR for mortality during index admission	OR for emergency readmission within 30 days	IRR for LOS of 30-day readmission
Univariate (unadjusted) (*n* = 9,267)	1.72 (1.42, 2.08), <0.001	2.16 (2.10, 2.22), <0.001	1.44 (1.00, 2.11), 0.05	2.31 (1.78, 3.00), <0.001	1.13 (1.05, 1.22), <0.001
Model 1^a^ (*n* = 9,267)	1.81 (1.50, 2.20), <0.001	2.27 (2.21, 2.34), <0.001	1.59 (1.08, 2.34), 0.02	2.46 (1.89, 3.21), <0.001	1.15 (1.07, 1.24), <0.001
Model 2^b^ (*n* = 9,262)	1.71 (1.41, 2.08), <0.001	2.23 (2.16, 2.29), <0.001	1.51 (1.03, 2.23), 0.04	2.43 (1.86, 3.18), <0.001	1.15 (1.07, 1.25), <0.001
Model 3^c^ (*n* = 9,262)	1.36 (1.10, 1.68), 0.01	1.78 (1.72, 1.83), <0.001	1.04 (0.68, 1.60), 0.85	2.05 (1.55, 2.72), <0.001	1.13 (1.04, 1.22), <0.001
Model 4^d^ (*n* = 9,262)	1.28 (1.03, 1.59), 0.04	1.27 (1.23, 1.32), <0.001	0.73 (0.45, 1.17), 0.19	1.79 (1.32, 3.43), <0.001	1.02 (0.92, 1.12), 0.78
Model 5^e^ (*n* = 9,262)		1.38 (1.33, 1.42), <0.001	0.87 (0.55, 1.38), 0.56	1.82 (1.35, 2.47), <0.001	0.97 (0.88, 1.07), 0.53

Abbreviations: IRR, incidence rate ratio; LOS, length of hospital stay; OR, odds ratio.

a Model 1 (adjusted for age and gender).

b Model 2 (adjusted for model 1 + ethnicity and IMD score).

c Model 3 (adjusted for model 2 + CVD hospital admission).

d Model 4 (adjusted for model 3 + physical disability related hospitalization).

e Model 5 (adjusted for model 4 + emergency vascular hospital admission).

Table 3 summarizes post-operative vascular surgical outcomes for patients with depression compared with the general population, categorized by type of vascular surgery (all adjustments are summarized in Supplementary Table S2). After adjusting for sociodemographic, physical, and CVD related confounding factors, patients with depression who had aortic/visceral endovascular surgery demonstrated significantly higher ORs for emergency readmissions within 30 days, had a 33% longer LOS for the index vascular hospital admission and also a 34% longer LOS for 30-day readmissions, as compared with the general population cohort. Patients with depression who had major open vascular surgery had 24% longer LOS for index vascular surgery compared with general population cohort. Patients with depression who received peripheral endovascular surgery had higher odds of both emergency hospital admission for the index vascular surgery and 30-day emergency readmission as well as a 47% longer LOS for the index vascular surgery; however, on the other hand the case group had a 23% shorter LOS for the 30-day hospital readmission, as compared with the general population cohort. Among patients with depression who underwent “other” vascular surgery, a 10% longer LOS for the index episode was found as compared with the general population cohort.

**Table 3.** Post-operative vascular surgery outcomes for patients with depression compared with those from the general population by type of vascular surgery OR/IRR (95% CI), *p*-Value.

	*Index vascular surgery hospitalization*			*Subsequent readmission*	
Type of vascular surgery	OR for emergency route of admission^**$**^	IRR for LOS*	OR for mortality during index admission*	OR for emergency readmission within 30 days*	IRR for LOS of 30-day readmission*
Aortic/visceral endovascular (*n* = 933)	1.36 (0.89, 2.84), 0.74	1.33 (1.18, 1.50), <0.001	0.94 (0.10, 8.54), 0.95	2.42 (1.02, 6.72), 0.05	1.34 (1.02, 2.03), 0.04
Major open vascular (*n* = 1,420)	1.04 (0.62, 1.78), 0.62	1.24 (1.15, 1.33), <0.001	0.89 (0.25, 3.09), 0.85	1.10 (0.48, 2.51), 0.82	0.79 (0.61, 1.02), 0.07
Peripheral endovascular (*n* = 3,984)	1.32 (1.01, 1.87), 0.05	1.47 (1.41, 1.53), <0.001	0.66 (0.37, 1.18), 0.16	2.32 (1.52, 3.55), <0.001	0.77 (0.67, 0.88), <0.001
Other (*n* = 2,925)	0.75 (0.49, 1.16), 0.20	1.10 (1.01, 1.20), 0.04	1.51 (0.39, 5.81), 0.55	1.47 (0.80, 2.70), 0.22	0.95 (0.74, 1.22), 0.69

Abbreviations: CI, confidence interval; IRR, incidence rate ratio; LOS, length of hospital stay; OR, odds ratio.

$ adjusted for age, gender, ethnicity, IMD score, CVD hospital admission, physical disability related hospitalisation

* Adjusted for age, gender ethnicity, IMD score, CVD hospital admission, physical disability related hospitalisation and emergency vascular hospital admission

Table 4 displays factors associated with post-operative health outcomes for patients with depression (univariate and multivariate models are available in Supplementary Table S3 for index hospitalizations and Supplementary Table S4 include univariate and multivariate models for 30-day hospital readmissions). Factors associated with emergency index hospitalization for vascular surgery included White ethnicity, having cognitive problems and having a previous hospital admission related to UTI. Factors predicting increased LOS for vascular surgery included admission via the emergency route, White ethnicity, higher neighborhood deprivation, having agitation problems, having cognitive problems, having daily living problems, having neurotic, stress-related, and somatoform disorders, receiving antipsychotics, antidepressants, and analgesic medication, having previous hospital admissions related to UTIs and previous CVD related hospital admissions related to arrhythmia, ischemia, and coronary heart disease, hypotension, diabetes, or heart failure. Factors associated with fewer number of days spent in hospital for vascular surgery included non-white ethnicity, hallucinations/delusions, substance abuse problems, and living conditions problems (as rated on the HoNOS), having dementia, receiving medication such as anxiolytics and hypnotics and antihypertensives, having previous hospital admissions due to syncope, osteoporosis, or hypercholesterolemia. Inpatient mortality was associated with admission via the emergency route and having previous hospital admissions relating to arrhythmia.

**Table 4.** Multivariate analysis showing predictors of health outcomes during vascular surgery spell and during emergency readmission to hospital within 30 days after discharge from hospital after vascular surgery spell for patients with depression.

		*Index vascular surgery hospitalization*		*Subsequent readmission*	
Predictors	OR for emergency route of admission during index hospitalization^a^ (*n* = 265)	IRR for LOS^a^ (*n* = 256)	OR for mortality during index admission^a^ (*n* = 443)	OR for emergency readmission within 30 days^a^ (*n* = 443)	IRR for LOS of 30-day readmission^a^ (*n* = 338)
Index admission-admitted to hospital via emergency route		5.09 (4.61, 5.63), <0.001	15.15 (3.48, 65.95), <0.001	1.99 (1.15, 3.43), 0.01	
Sociodemographic characteristics					
10 year increase in age at hospital admission					
Male gender			0.36 (0.15, 0.88), 0.01		1.70 (1.40, 2.08), <0.001
Non-white ethnicity	0.51 (0.30, 0.86), 0.01	0.80 (0.73, 0.88), <0.001			
10-unit increase in IMD		1.05 (1.01, 1.09), 0.02			
HoNoS problems					
Agitation problems		1.15 (1.00, 1.32), 0.05			1.61 (1.01, 2.57), 0.05
Self-injury problems					0.41 (0.3, 0.58), <0.001
Substance abuse problems		0.84 (0.72, 0.99), 0.03			
Cognitive problems	1.99 (1.04, 3.80), 0.04	1.47 (1.33, 1.62), <0.001			
Hallucinations		0.50 (0.40, 0.61), <0.001			
Depressed					1.67 (1.35, 2.06), <0.001
Daily living problems		1.45 (1.34, 1.58), <0.001			
Living conditions problems		0.83 (0.74, 0.93), <0.001			
Occupational problems					2.30 (1.8, 2.95), <0.001
Psychiatric diagnosis					
Mental and behavioral disorders due to psychoactive substance use (F10–F19)				2.52 (1.01, 6.50), 0.05	0.34 (0.23, 0.50), <0.001
Dementia		0.67 (0.57, 0.78), <0.001			
Neurotic, stress-related and somatoform disorders (F40–F48)		1.33 (1.16, 1.52), <0.001			
Disorders of adult personality and behavior (F60–F69)					0.12 (0.04, 0.31), <0.001
Psychiatric medication before vascular surgery					
Antipsychotics		1.15 (1.02, 1.29), 0.02			
Antidepressants		1.11 (1.01, 1.22), 0.03			1.98 (1.62, 2.41), <0.001
Anxiolytics and hypnotics		0.67 (0.59, 0.75), <0.001			
Physical health medication					
Analgesics		1.21 (1.09, 1.33), <0.001			
Antihypertensives		0.78 (0.70, 0.86), <0.001			
Previous physical disability-related admissions					
Syncope and collapse		0.88 (0.81, 0.95), <0.001		1.74 (1.02, 2.98), 0.04	0.62 (0.52, 0.74), <0.001
Osteoporosis		0.72 (0.64, 0.82), <0.001			
UTIs	2.59 (1.39, 4.83), <0.001	1.41 (1.29, 1.54), <0.001			2.03 (1.67, 2.48), <0.001
Previous CVD hospital admissions					
Arrhythmia		3.54 (1.59, 7.86), <0.001	5.29 (2.12, 13.21), <0.001		
Ischemia and coronary heart disease		1.65 (1.49, 1.82), <0.001			
Hypertension			0.29 (0.11, 0.71), <0.001		
Hypotension		1.42 (1.27, 1.58), <0.001	2.45 (1.02, 6.58), 0.04		
Diabetes		1.15 (1.07, 1.25), <0.001			
Heart failure		1.86 (1.70, 2.04), <0.001			0.68 (0.52, 0.89), 0.01
Hypercholesterolemia		0.69 (0.62, 0.76), <0.001		2.39 (1.39, 4.10), <0.001	

Abbreviations: CVD, cardiovascular disease; IMD, index of multiple deprivation; IRR, incidence rate ratio; LOS, length of hospital stay; OR, odds ratio; UTI, urinary tract infections.

a Multivariate analysis adjusted for each other variables.

Considering subsequent hospitalization (Table 4) emergency hospital readmissions within 30 days of discharge following vascular surgery were associated with emergency route index admission, presence of mental and behavioral disorders due to psychoactive substance use and previous hospital admissions due to syncope and collapse or hypercholesterolemia. LOS for emergency 30-day readmissions was positively associated with male gender, agitation problems, being depressed, having occupational problems, taking antidepressants, and previous hospital admissions related to UTI’s, while it was negatively associated with self-injury problems, mental, and behavioral problems due to psychoactive substance use, disorders of adult personality, and behavior, and having previous hospital admissions relating to syncope and heart failure.

## Discussion

This study investigated admission rates and post-operative health outcomes of vascular surgery for patients with depression compared to the general population, and to estimate key predictors of these outcomes. The primary investigation found a significantly raised SAR for emergency as well as elective hospital admissions for patients with diagnosed depression compared to the general population living in the same catchment area, therefore suggesting a greater risk to cardiovascular health for patients with depression, consistent with previous research [1, 2, 33]. Patients with depression also had a higher likelihood of emergency rather than elective index admission, a longer index hospital LOS for vascular surgery and a higher risk of 30-day emergency hospital readmission, after taking into account a wide range of potential confounding factors.

Considering health outcomes measured at the index hospitalization, patients with depression were significantly more likely to have been admitted via an emergency route and then also stayed in hospital for longer. However, although inpatient mortality rates during index hospitalization were greater for patients with depression, this did not differ significantly to general public counterparts in fully adjusted analyses. This suggests that higher post-operative mortality for patients with depression is at least partly accounted for by the physical health comorbidity present in this population, whereas this does not account for emergency admission and LOS outcomes. Determining predictors of each outcome can be helpful in identifying underlying correlates that may explain different outcome profiles. Predictors including white ethnicity, having cognitive problems and having previous UTI related hospital admissions were associated with both emergency index admission and index hospital LOS. It is plausible that depression is associated with cognitive problems and poorer lifestyle habits, resulting in UTI’s, which might explain the increased risk for vascular surgery outcomes. Furthermore, factors associated with inpatient mortality include emergency admission and previous arrhythmia-related admissions, which were also associated with index LOS.

Considering further hospitalization, patients with depression were significantly more likely to have emergency readmissions, although the LOS for these readmission episodes did not differ significantly between patients with depression and the general population in fully adjusted models, thus suggesting that comorbidity might account for observed differences, similar to index inpatient mortality as an outcome. Previous research suggests that risk factors associated with 30-day readmission due to infections following lower extremity vascular procedures include longer LOS, previous infections, anemia, and included UTI infections [34]. Findings in our cohort of people with diagnosed depression indicate that emergency 30-day hospital readmission is associated with emergency index admission, and that longer readmission LOS is associated previous UTI-related admissions, supporting previous findings. Diabetes diagnoses at index hospitalization was associated with longer index LOS in the present study, yet predicted emergency 30-day readmission in previous findings [34]. Differences in predictors for LOS between the index and readmission hospitalizations warrant further research, ideally with more information than could be gathered here on inpatient pathways of care and on aftercare provided in the periods between hospitalization episodes.

The impact of depression on vascular surgery outcomes may also depend on the type of vascular procedure. In the present study, we categorized four broad types of vascular surgery: aortic/visceral endovascular, major open vascular, peripheral endovascular, and other vascular. Patients with depression did not differ from the general population in the types of vascular procedures that were carried out; however, in terms of post-operative health outcomes, patients with depression undergoing endovascular procedures had longer index LOS, readmission LOS, and emergency readmission. In comparison, although index LOS was significantly longer for patients with depression, other outcome risks were not as pronounced for major open vascular procedures. The key difference between these types of procedures is that the endovascular procedures are markedly less invasive than the major open procedures. Due to the more complex nature of the surgeries, major open procedures inherently pose a greater risk for physiological insult, resulting in longer LOS, greater post-operative complications or pain, and other challenges. As the standard recovery processes for these procedures are generally more complex, the implications for patients with depression may not be as observable, or at least not in the current study sample. For less invasive procedures, post-operative recovery is not as debilitating and so patients may be recognized as safe for discharge much sooner than for major open procedures. In these instances, the detrimental implications of depression may be more discernible following surgery. For example, post-operative pain, decreased treatment adherence, and risk lifestyles [8–14] might result in the observed increased likelihood of emergency readmission and longer LOS. The implications for index and readmission emergency hospitalization, as well as index and readmission LOS should definitely be considered for patients with depression undergoing any type of vascular surgery.

This study does have several limitations. First, our findings are observational and drawn from routine electronic health records and so causal inferences cannot conclusively be drawn. Second, data were not available on detailed post-operative monitoring and care, although previous research has reported higher rates of hospital readmissions following less adequate inpatient care and post-operative complications [35, 36]. Similarly, volume of surgeries and surgeon training also have implications for patient prognosis [37] but could not be taken into account. Third, findings on outcomes and predictors were limited to patients from a single site and catchment, and further evaluation is required to establish wider generalizability. Finally, data extracted had some limitations in scope; for example, we did not collect information on different types of antihypertensives.

Overall, the naturalistic, large scale data used in this study, based on an ethnically diverse, varied population were key strengths over some of the more selected samples investigated to date, and to our knowledge, this study is the first of its kind to assess the associations between mental health and vascular surgery outcomes on this scale. Further research, particularly involving more detailed information on inpatient and outpatient care, is needed to determine why these risks to patients with depression might have been observed, whether these risks actually differ between types of vascular procedures and if these findings are replicated with other mental health conditions. The findings have potentially major implications for pre-operative assessments in patients being selected for vascular surgery or requiring this as an emergency. In this respect, we recommend that patients are screened for depression, particularly if the patient is undergoing endovascular procedures, that the implications for their recovery and prognosis are considered, and that interventions are developed and evaluated to improve equality of care and outcomes.

## Data Availability

No additional data are available.

## Supplementary material

For supplementary material accompanying this paper visit https://doi.org/10.1192/j.eurpsy.2021.2. click here to view supplementary material

## References

[1] Carney RM, Freedland KE. Depression, mortality, and medical morbidity in patients with coronary heart disease. Biol Psychiatry. 2003;54(3):241–247. 12893100 10.1016/s0006-3223(03)00111-2

[2] Wattanakit K, Folsom AR, Selvin E, Weatherley BD, Pankow JS, Brancati FL, et al. Risk factors for peripheral arterial disease incidence in persons with diabetes: the Atherosclerosis Risk in Communities (ARIC) Study. Atherosclerosis. 2005;180(2):389–397. 15910867 10.1016/j.atherosclerosis.2004.11.024

[3] Harris TB, Ferrucci L, Tracy RP, Corti MC, Wacholder S, Ettinger Jr WH, et al. Associations of elevated interleukin-6 and C-reactive protein levels with mortality in the elderly. Am J Med. 1999;106(5):506–12. 10335721 10.1016/s0002-9343(99)00066-2

[4] Nikkheslat N, Zunszain PA, Horowitz MA, Barbosa IG, Parker JA, Myint AM, et al. Insufficient glucocorticoid signaling and elevated inflammation in coronary heart disease patients with comorbid depression. Brain Behav Immunity. 2015;48:8–18. 10.1016/j.bbi.2015.02.002 25683698

[5] Criqui MH, Langer RD, Fronek A, Feigelson HS, Klauber MR, McCann TJ, et al. Mortality over a period of 10 years in patients with peripheral arterial disease. New Engl J Med. 1992;326(6):381–386. 1729621 10.1056/NEJM199202063260605

[6] Hernandez NV, Ramirez JL, Khetani SA, Spaulding KA, Gasper WJ, Hiramoto J, et al. Depression severity is associated with increased inflammation in veterans with peripheral artery disease. Vascular Med. 2018;23(5):445–453. 10.1177/1358863X18787640 30035700

[7] Ramirez JL, Grenon SM. Depression and peripheral artery disease: why we should care and what we can do. CVIR Endovascular. 2018;1(1):1–2. 30652146 10.1186/s42155-018-0017-1 PMC6319506

[8] De Cosmo G, Congedo E, Lai C, Primieri P, Dottarelli A, Aceto P. Preoperative psychologic and demographic predictors of pain perception and tramadol consumption using intravenous patient-controlled analgesia. Clin J Pain. 2008;24(5):399–405. 18496304 10.1097/AJP.0b013e3181671a08

[9] Gerbershagen HJ, Aduckathil S, van Wijck AJ, Peelen LM, Kalkman CJ, Meissner W. Pain intensity on the first day after surgery: a prospective cohort study comparing 179 surgical procedures. Anesthesiol: J Am Soc Anesthesiol. 2013;118(4):934–944. 10.1097/ALN.0b013e31828866b3 23392233

[10] Joshi GP, Kehlet H. Procedure-specific pain management: the road to improve postsurgical pain management? Anesthesiol: J Am Soc Anesthesiol. 2013;118(4):780–782. 10.1097/ALN.0b013e31828866e1 23388191

[11] Ghoneim MM, O’Hara MW. Depression and postoperative complications: an overview. BMC Surg. 2016;16(1):5. 26830195 10.1186/s12893-016-0120-y PMC4736276

[12] Abrams TE, Vaughan-Sarrazin M, Rosenthal GE. Influence of psychiatric comorbidity on surgical mortality. Arch Surg. 2010;145(10):947–953. 20956762 10.1001/archsurg.2010.190

[13] Rieckmann N, Kronish IM, Haas D, Gerin W, Chaplin WF, Burg MM, et al. Persistent depressive symptoms lower aspirin adherence after acute coronary syndromes. Am Heart J. 2006;152(5):922–927. 17070160 10.1016/j.ahj.2006.05.014

[14] van Gool CH, Kempen GI, Bosma H, van Boxtel MP, Jolles J, van Eijk JT. Associations between lifestyle and depressed mood: longitudinal results from the Maastricht aging study. Am J Publ Health. 2007;97(5):887–894. 10.2105/AJPH.2004.053199 PMC1854865 16735630

[15] Arya S, Lee S, Zahner GJ, Cohen BE, Hiramoto J, Wolkowitz OM, et al. The association of comorbid depression with mortality and amputation in veterans with peripheral artery disease. J Vasc Surg. 2018;68(2):536–545. 29588133 10.1016/j.jvs.2017.10.092 PMC6057818

[16] Cherr GS, Wang J, Zimmerman PM, Dosluoglu HH. Depression is associated with worse patency and recurrent leg symptoms after lower extremity revascularization. J Vasc Surg. 2007;45(4):744–750. 17303367 10.1016/j.jvs.2006.11.057

[17] Cherr GS, Zimmerman PM, Wang J, Dosluoglu HH. Patients with depression are at increased risk for secondary cardiovascular events after lower extremity revascularization. J Gen Internal Med. 2008;23(5):629–634. 18299940 10.1007/s11606-008-0560-x PMC2324156

[18] McDermott MM, Guralnik JM, Tian L, Kibbe MR, Ferrucci L, Zhao L, et al. Incidence and prognostic significance of depressive symptoms in peripheral artery disease. J Am Heart Assoc. 2016;5(3):e002959. 26994131 10.1161/JAHA.115.002959 PMC4943270

[19] Copeland LA, Sako EY, Zeber JE, Pugh MJ, Wang CP, MacCarthy AA, et al. Mortality after cardiac or vascular operations by preexisting serious mental illness status in the Veterans Health Administration. Gen Hospital Psychiatry. 2014;36(5):502–508. 10.1016/j.genhosppsych.2014.04.003 24957928

[20] Dao TK, Chu D, Springer J, Gopaldas RR, Menefee DS, Anderson T, et al. Clinical depression, posttraumatic stress disorder, and comorbid depression and posttraumatic stress disorder as risk factors for in-hospital mortality after coronary artery bypass grafting surgery. J Thoracic Cardiovasc Surg. 2010;140(3):606–610. 10.1016/j.jtcvs.2009.10.046 20074753

[21] Takagi H, Ando T, Umemoto T. ALICE (all-literature investigation of cardiovascular evidence) group. Perioperative depression or anxiety and postoperative mortality in cardiac surgery: a systematic review and meta-analysis. Heart Vessels. 2017;32(12):1458–1468. 28702898 10.1007/s00380-017-1022-3

[22] Joseph HK, Whitcomb J, Taylor W. Effect of anxiety on individuals and caregivers after coronary artery bypass grafting surgery: a review of the literature. Dimens Crit Care Nurs. 2015;34(5):285–288. 26244244 10.1097/DCC.0000000000000137

[23] Copeland LA, Zeber JE, Pugh MJ, Mortensen EM, Restrepo MI, Lawrence VA. Postoperative complications in the seriously mentally ill: a systematic review of the literature. Ann Surg. 2008;248(1):31–38. 18580204 10.1097/SLA.0b013e3181724f25

[24] Lee DS, Chiu M, Manuel DG, Tu K, Wang X, Austin PC, et al. Trends in risk factors for cardiovascular disease in Canada: temporal, socio-demographic and geographic factors. CMAJ. 2009;181(3–4):E55–E66. 19620271 10.1503/cmaj.081629 PMC2717674

[25] Hannan EL, Racz MJ, Walford G, Ryan TJ, Isom OW, Bennett E, et al. Predictors of readmission for complications of coronary artery bypass graft surgery. JAMA. 2003;290(6):773–780. 12915430 10.1001/jama.290.6.773

[26] Desbois AC, Cacoub P. Cannabis-associated arterial disease. Ann Vasc Surg. 2013;27(7):996–1005. 23850313 10.1016/j.avsg.2013.01.002

[27] McGuinness BR, Goel A, Elias F, Rapanos T, Mittleman M, Ladha KS. Cannabis use disorder and perioperative outcomes in vascular surgery. J Vasc Surg. 2020;72(1):e171–e172. 10.1016/j.jvs.2020.07.094 32861869

[28] Dewan KC, Dewan KS, Idrees JJ, Navale SM, Rosinski BF, Svensson LG, et al. Trends and outcomes of cardiovascular surgery in patients with opioid use disorders. JAMA Surg. 2019;154(3):232–240. 30516807 10.1001/jamasurg.2018.4608 PMC6439637

[29] National GC. Preoperative tests (update): routine preoperative tests for elective surgery. 27077168

[30] Perera G, Broadbent M, Callard F, Chang CK, Downs J, Dutta R, et al. Cohort profile of the South London and Maudsley NHS Foundation Trust Biomedical Research Centre (SLaM BRC) case register: current status and recent enhancement of an electronic mental health record-derived data resource. BMJ Open. 2016;6(3) e008721. 10.1136/bmjopen-2015-008721 PMC4785292 26932138

[31] Fernandes AC, Cloete D, Broadbent MT, Hayes RD, Chang CK, Jackson RG, et al. Development and evaluation of a de-identification procedure for a case register sourced from mental health electronic records. BMC Med Inform Decis Mak. 2013;13(1):71. 23842533 10.1186/1472-6947-13-71 PMC3751474

[32] NHS Data dictionary accessed on 10th June 2020. https://www.datadictionary.nhs.uk/data_dictionary/attributes/a/add/admission_method_de.asp?shownav=1?query=%22method+of+admission%22&rank=85.71428&shownav=1.

[33] Cameron AC, Trivedi PK. Microeconometrics using stata. College Station, TX: Stata Press, 2009.

[34] Melvin JC, Smith JB, Kruse RL, Vogel TR. Risk factors for 30-day hospital re-admission with an infectious complication after lower-extremity vascular procedures. Surg Infect. 2017;18(3):319–326. 10.1089/sur.2016.234 PMC5796593 28177854

[35] Ashton CM, Del Junco DJ, Souchek J, Wray NP, Mansyur CL. The association between the quality of inpatient care and early readmission: a meta-analysis of the evidence. Med Care. 1997;1:1044–1059. 10.1097/00005650-199710000-00006 9338530

[36] Encinosa WE, Bernard DM, Chen CC, Steiner CA. Healthcare utilization and outcomes after bariatric surgery. Med Care. 2006;1:706–712. 10.1097/01.mlr.0000220833.89050.ed 16862031

[37] Pearce WH, Parker MA, Feinglass J, Ujiki M, Manheim LM. The importance of surgeon volume and training in outcomes for vascular surgical procedures. J Vasc Surg. 1999;29(5):768–778. 10231626 10.1016/s0741-5214(99)70202-8

