# Supplementary material for: A prospective investigation of depression and adverse outcomes in patients undergoing vascular surgical interventions: A retrospective cohort study using a large mental health database in South London
Source: Eur Psychiatry. 2021 Jan 18;64(1):e13. doi: 10.1192/j.eurpsy.2021.2 (PMC8057466; doi:10.1192/j.eurpsy.2021.2)
Supplement: Supplementary file 1 [file epasup.zip › S092493382100002Xsup001.docx]

**Supplementary table 1: OPCS Classification of Interventions and Procedures version 4 codes for vascular surgical procedures included in the study**

| **OPCS-4 Code** | **OPCS-4 Description** | **Type of vascular surgery** |
| --- | --- | --- |
| L192 | REPLACEMENT OF ANEURYSMAL SEGMENT OF THORACIC AORTA BY ANAST | Major open |
| L193 | REPLACEMENT OF ANEURYSMAL SEGMENT OF SUPRARENAL ABDOMINAL AO | Major open |
| L194 | REPLACEMENT OF ANEURYSMAL SEGMENT OF INFRARENAL ABDOMINAL AO | Major open |
| L195 | REPLACEMENT OF ANEURYSMAL SEGMENT OF ABDOMINAL AORTA BY ANAS | Major open |
| L196 | REPLACEMENT OF ANEURYSMAL BIFURCATION OF AORTA BY ANASTOMOSI | Major open |
| L198 | OTHER REPLACEMENT OF ANEURYSMAL SEGMENT OF AORTA (O) | Major open |
| L199 | OTHER REPLACEMENT OF ANEURYSMAL SEGMENT OF AORTA (U) | Major open |
| L213 | BYPASS OF SEGMENT OF SUPRARENAL ABDOMINAL AORTA BY ANASTOMOS | Major open |
| L214 | BYPASS OF SEGMENT OF INFRARENAL ABDOMINAL AORTA BY ANASTOMOS | Major open |
| L215 | BYPASS OF SEGMENT OF ABDOMINAL AORTA BY ANASTOMOSIS OF AORTA | Major open |
| L216 | BYPASS OF BIFURCATION OF AORTA BY ANASTOMOSIS OF AORTA TO IL | Major open |
| L218 | OTHER BYPASS OF SEGMENT OF AORTA (O) | Major open |
| L219 | OTHER BYPASS OF SEGMENT OF AORTA (U) | Major open |
| L221 | REVISION OF PROSTHESIS OF THORACIC AORTA | Major open |
| L222 | REVISION OF PROSTHESIS OF BIFURCATION OF AORTA | Major open |
| L223 | REVISION OF PROSTHESIS OF ABDOMINAL AORTA NEC | Major open |
| L224 | REMOVAL OF PROSTHESIS FROM AORTA | Major open |
| L228 | ATTENTION TO PROSTHESIS OF AORTA (O) | Major open |
| L229 | ATTENTION TO PROSTHESIS OF AORTA (U) | Major open |
| L231 | PLASTIC REPAIR OF AORTA AND END TO END ANASTOMOSIS OF AORTA | Major open |
| L232 | PLASTIC REPAIR OF AORTA USING SUBCLAVIAN FLAP | Major open |
| L233 | PLASTIC REPAIR OF AORTA USING PATCH GRAFT | Major open |
| L234 | RELEASE OF VASCULAR RING OF AORTA | Major open |
| L235 | REVISION OF PLASTIC REPAIR OF AORTA | Major open |
| L238 | PLASTIC REPAIR OF AORTA (O) | Major open |
| L239 | PLASTIC REPAIR OF AORTA (U) | Major open |
| L251 | ENDARTERECTOMY OF AORTA AND PATCH REPAIR OF AORTA | Major open |
| L252 | ENDARTERECTOMY OF AORTA NEC | Major open |
| L253 | OPEN EMBOLECTOMY OF BIFURCATION OF AORTA | Major open |
| L254 | OPERATIONS ON ANEURYSM OF AORTA NEC | Major open |
| L255 | OPERATIONS ON AORTIC BODY | Major open |
| L258 | OTHER OPEN OPERATIONS ON AORTA (O) | Major open |
| L259 | OTHER OPEN OPERATIONS ON AORTA (U) | Major open |
| L291 | REPLACEMENT OF CAROTID ARTERY USING GRAFT | Major open |
| L292 | INTRACRANIAL BYPASS TO CAROTID ARTERY | Major open |
| L293 | BYPASS TO CAROTID ARTERY NEC | Major open |
| L294 | ENDARTERECTOMY OF CAROTID ARTERY AND PATCH REPAIR OF CAROTID | Major open |
| L295 | ENDARTERECTOMY OF CAROTID ARTERY NEC | Major open |
| L298 | RECONSTRUCTION OF CAROTID ARTERY (O) | Major open |
| L299 | RECONSTRUCTION OF CAROTID ARTERY (U) | Major open |
| L301 | REPAIR OF CAROTID ARTERY NEC | Major open |
| L302 | LIGATION OF CAROTID ARTERY | Major open |
| L303 | OPEN EMBOLECTOMY OF CAROTID ARTERY | Major open |
| L304 | OPERATIONS ON ANEURYSM OF CAROTID ARTERY | Major open |
| L305 | OPERATIONS ON CAROTID BODY | Major open |
| L308 | OTHER OPEN OPERATIONS ON CAROTID ARTERY (O) | Major open |
| L309 | OTHER OPEN OPERATIONS ON CAROTID ARTERY (U) | Major open |
| L373 | ENDARTERECTOMY OF SUBCLAVIAN ARTERY AND PATCH REPAIR OF SUBC | Major open |
| L374 | ENDARTERECTOMY OF SUBCLAVIAN ARTERY NEC | Major open |
| L378 | RECONSTRUCTION OF SUBCLAVIAN ARTERY (O) | Major open |
| L379 | RECONSTRUCTION OF SUBCLAVIAN ARTERY (U) | Major open |
| L381 | REPAIR OF SUBCLAVIAN ARTERY NEC | Major open |
| L382 | LIGATION OF SUBCLAVIAN ARTERY | Major open |
| L384 | OPERATIONS ON ANEURYSM OF SUBCLAVIAN ARTERY | Major open |
| L388 | OTHER OPEN OPERATIONS ON SUBCLAVIAN ARTERY (O) | Major open |
| L389 | OTHER OPEN OPERATIONS ON SUBCLAVIAN ARTERY (U) | Major open |
| L411 | PLASTIC REPAIR OF RENAL ARTERY AND END TO END ANASTOMOSIS | Major open |
| L413 | REPLANTATION OF RENAL ARTERY | Major open |
| L412 | BYPASS OF RENAL ARTERY | Major open |
| L414 | ENDARTERECTOMY OF RENAL ARTERY | Major open |
| L415 | TRANSLOCATION OF BRANCH OF RENAL ARTERY | Major open |
| L418 | RECONSTRUCTION OF RENAL ARTERY (O) | Major open |
| L419 | RECONSTRUCTION OF RENAL ARTERY (U) | Major open |
| L421 | OPEN EMBOLECTOMY OF RENAL ARTERY | Major open |
| L422 | OPEN EMBOLISATION OF RENAL ARTERY | Major open |
| L423 | LIGATION OF RENAL ARTERY | Major open |
| L424 | OPERATIONS ON ANEURYSM OF RENAL ARTERY | Major open |
| L428 | OTHER OPEN OPERATIONS ON RENAL ARTERY (O) | Major open |
| L429 | OTHER OPEN OPERATIONS ON RENAL ARTERY (U) | Major open |
| L451 | BYPASS OF VISCERAL BRANCH OF ABDOMINAL AORTA NEC | Major open |
| L452 | REPLANTATION OF VISCERAL BRANCH OF ABDOMINAL AORTA NEC | Major open |
| L453 | ENDARTERECTOMY OF VISCERAL BRANCH OF ABDOMINAL AORTA AND PAT | Major open |
| L454 | ENDARTERECTOMY OF VISCERAL BRANCH OF ABDOMINAL AORTA NEC | Major open |
| L458 | RECONSTRUCTION OF OTHER VISCERAL BRANCH OF ABDOMINAL AO (O) | Major open |
| L459 | RECONSTRUCTION OF OTHER VISCERAL BRANCH OF ABDOMINAL AO (U) | Major open |
| L461 | OPEN EMBOLECTOMY OF VISCERAL BRANCH OF ABDOMINAL AORTA NEC | Major open |
| L462 | OPEN EMBOLISATION OF VISCERAL BRANCH OF ABOF ABDOMINAL AORTA | Major open |
| L463 | LIGATION OF VISCERAL BRANCH OF ABDOMINAL AORTA NEC | Major open |
| L464 | OPS ON ANEURYSM OF VISCERAL | Major open |
| L468 | OTHER OPEN OPS ON OTHER VISCERAL BRANCH/ABDOMINAL AORTA (O) | Major open |
| L469 | OTHER OPEN OPS ON OTHER VISCERAL BRANCH/ABDOMINAL AORTA (U) | Major open |
| L491 | REPLACEMENT/ANEURYSMAL COMMON IL.ART.BY ANASTOMOSIS/AORTA TO | Major open |
| L492 | REPLACEMENT/ANEURYSMAL IL.ART.BY ANASTOMOSIS/AORTA TO EXTERN | Major open |
| L493 | REPLACEMENT/ANEURYSMAL ART.OF LEG BY ANASTOMOSIS/AORTA TO CO | Major open |
| L494 | REPLACEMENT/ANEURYSMAL ART.OF LEG BY ANASTOMOSIS/AORTA TO SU | Major open |
| L495 | REPLACEMENT/ANEURYSMAL IL.ART.BY ANASTOMOSIS/ILIAC ARTERY TO | Major open |
| L496 | REPLACEMENT/ANEURYSMAL ART OF LEG BY ANASTOMOSIS/ILIAC ARTER | Major open |
| L498 | OTHER REPLACEMENT OF ANEURYSMAL ILIAC ARTERY (O) | Major open |
| L499 | OTHER REPLACEMENT OF ANEURYSMAL ILIAC ARTERY (U) | Major open |
| L511 | BYPASS OF COMMON ILIAC ARTERY BY ANASTOMOSIS OF AORTA TO COM | Major open |
| L512 | BYPASS OF ILIAC ARTERY BY ANASTOMOSIS OF AORTA TO EXTERNAL I | Major open |
| L513 | BYPASS OF ARTERY OF LEG BY ANASTOMOSIS OF AORTA TO COMMON FE | Major open |
| L514 | BYPASS OF ARTERY OF LEG BY ANASTOMOSIS OF AORTA TO DEEP FEMO | Major open |
| L515 | BYPASS OF ILIAC ARTERY BY ANASTOMOSIS OF ILIAC ARTERY TO ILI | Major open |
| L516 | BYPASS OF ARTERY OF LEG BY ANASTOMOSIS OF ILIAC ARTERY TO FE | Major open |
| L518 | OTHER BYPASS OF ILIAC ARTERY (O) | Major open |
| L519 | OTHER BYPASS OF ILIAC ARTERY (U) | Major open |
| L521 | ENDARTERECTOMY OF ILIAC ARTERY AND PATCH REPAIR OF ILIAC ART | Major open |
| L522 | ENDARTERECTOMY OF ILIAC ARTERY NEC | Major open |
| L528 | RECONSTRUCTION OF ILIAC ARTERY (O) | Major open |
| L529 | RECONSTRUCTION OF ILIAC ARTERY (U) | Major open |
| L531 | REPAIR OF ILIAC ARTERY NEC | Major open |
| L533 | OPERATIONS ON ANEURYSM OF ILIAC ARTERY NEC | Major open |
| L538 | OTHER OPEN OPERATIONS ON ILIAC ARTERY (O) | Major open |
| L539 | OTHER OPEN OPERATIONS ON ILIAC ARTERY (U) | Major open |
| L571 | REP/ANEUR.FEM.ART.BY ANASTOMOSIS OF FEM.ART.TO FEM.ART.NEC | Major open |
| L572 | REP/ANEUR.FEM.ART/ANASTOMOSIS/FEM.ART.TO POP.ART.USING PROST | Major open |
| L573 | REP/ANEUR.FEM.ART/ANASTOMOSIS/FEM.ART.TO POP.ART.USING VEIN | Major open |
| L574 | REP/ANEUR.FEM.ART/ANASTOMOSIS/FEM.ART.TO TIB.ART.USING PROST | Major open |
| L575 | REP/ANEUR.FEM.ART/ANASTOMOSIS/FEM.ART.TO TIB.ART.USING VEIN | Major open |
| L576 | REP/ANEUR.FEM.ART/ANASTOMOSIS/FEM.ART.TO PER.ART.USING PROST | Major open |
| L577 | REP/ANEUR.FEM.ART/ANASTOMOSIS/FEM.ART.TO PER.ART.USING VEIN | Major open |
| L578 | OTHER REPLACEMENT OF ANEURYSMAL FEMORAL ARTERY (O) | Major open |
| L579 | OTHER REPLACEMENT OF ANEURYSMAL FEMORAL ARTERY (U) | Major open |
| L591 | BYPASS OF FEMORAL ARTERY BY ANASTOMOSIS OF FEMORAL ARTERY TO | Major open |
| L592 | BYPASS/FEM.ART.BY ANASTOMOSIS OF FEM.ART.TO POP.ART.USING PR | Major open |
| L593 | BYPASS/FEM.ART.BY ANASTOMOSIS OF FEM.ART.TO POP.ART.USING VE | Major open |
| L594 | BYPASS/FEM.ART.BY ANASTOMOSIS OF FEM.ART.TO TIB.ART.USING PR | Major open |
| L595 | BYPASS/FEM.ART.BY ANASTOMOSIS OF FEM.ART.TO TIB.ART.USING VE | Major open |
| L596 | BYPASS/FEM.ART.BY ANASTOMOSIS OF FEM.ART.TO PER.ART.USING PR | Major open |
| L597 | BYPASS/FEM.ART.BY ANASTOMOSIS OF FEM.ART.TO PER.ART.USING VE | Major open |
| L598 | OTHER BYPASS OF FEMORAL ARTERY (O) | Major open |
| L599 | OTHER BYPASS OF FEMORAL ARTERY (U) | Major open |
| L601 | ENDARTERECTOMY OF FEMORAL ARTERY AND PATCH REPAIR OF FEMORAL | Major open |
| L602 | ENDARTERECTOMY OF FEMORAL ARTERY NEC | Major open |
| L603 | PROFUNDOPLASTY OF FEMORAL ARTERY AND PATCH REPAIR OF DEEP FE | Major open |
| L604 | PROFUNDOPLASTY OF FEMORAL ARTERY NEC | Major open |
| L608 | RECONSTRUCTION OF FEMORAL ARTERY (O) | Major open |
| L609 | RECONSTRUCTION OF FEMORAL ARTERY (U) | Major open |
| L621 | REPAIR OF FEMORAL ARTERY NEC | Major open |
| L623 | LIGATION OF ANEURYSM OF POPLITEAL ARTERY | Major open |
| L624 | OPERATIONS ON ANEURYSM OF FEMORAL ARTERY NEC | Major open |
| L628 | OTHER OPEN OPERATIONS ON FEMORAL ARTERY (O) | Major open |
| L629 | OTHER OPEN OPERATIONS ON FEMORAL ARTERY (U) | Major open |
| L651 | REVISION OF RECONSTRUCTION INVOLVING AORTA | Major open |
| L652 | REVISION OF RECONSTRUCTION INVOLVING ILIAC ARTERY | Major open |
| L653 | REVISION OF RECONSTRUCTION INVOLVING FEMORAL ARTERY | Major open |
| L658 | REVISION OF RECONSTRUCTION OF ARTERY (O) | Major open |
| L659 | REVISION OF RECONSTRUCTION OF ARTERY (U) | Major open |
| L681 | ENDARTERECTOMY AND PATCH REPAIR OF ARTERY NEC | Major open |
| L682 | ENDARTERECTOMY NEC | Major open |
| L683 | REPAIR OF ARTERY USING PROSTHESIS NEC | Major open |
| L684 | REPAIR OF ARTERY USING VEIN GRAFT NEC | Major open |
| L705 | OPERATIONS ON ANEURYSM OF ARTERY NEC | Major open |
| L261 | PERCUTANEOUS TRANSLUMINAL BALLOON ANGIOPLASTY OF AORTA | Aortic/visceral endovascular |
| L262 | PERCUTANEOUS TRANSLUMINAL ANGIOPLASTY OF AORTA NEC | Aortic/visceral endovascular |
| L263 | PERCUTANEOUS TRANSLUMINAL EMBOLECTOMY OF BIFURCATION OF AORT | Aortic/visceral endovascular |
| L264 | AORTOGRAPHY | Aortic/visceral endovascular |
| L268 | TRANSLUMINAL OPERATIONS ON AORTA (O) | Aortic/visceral endovascular |
| L269 | TRANSLUMINAL OPERATIONS ON AORTA (U) | Aortic/visceral endovascular |
| L431 | PERCUTANEOUS TRANSLUMINAL ANGIOPLASTY OF RENAL ARTERY | Aortic/visceral endovascular |
| L432 | PERCUTANEOUS TRANSLUMINAL EMBOLECTOMY OF RENAL ARTERY | Aortic/visceral endovascular |
| L433 | PERCUTANEOUS TRANSLUMINAL EMBOLISATION OF RENAL ARTERY | Aortic/visceral endovascular |
| L434 | ARTERIOGRAPHY OF RENAL ARTERY | Aortic/visceral endovascular |
| L438 | TRANSLUMINAL OPERATIONS ON RENAL ARTERY (O) | Aortic/visceral endovascular |
| L439 | TRANSLUMINAL OPERATIONS ON RENAL ARTERY (U) | Aortic/visceral endovascular |
| L471 | PERCUTANEOUS TRANSLUMINAL ANGIOPLASTY OF VISCERAL BRANCH OF | Aortic/visceral endovascular |
| L472 | PERCUTANEOUS TRANSLUMINAL EMBOLISATION OF VISCERAL BRANCH | Aortic/visceral endovascular |
| L473 | ARTERIOGRAPHY OF VISCERAL BRANCH OF ABDOMINAL AORTA NEC | Aortic/visceral endovascular |
| L478 | TRANSLUMINAL OPS ON OTHER VISCERAL BRANCH/ABDOMINAL AOR (O) | Aortic/visceral endovascular |
| L479 | TRANSLUMINAL OPS ON OTHER VISCERAL BRANCH/ABDOMINAL AOR (U) | Aortic/visceral endovascular |
| L311 | PERCUTANEOUS TRANSLUMINAL ANGIOPLASTY OF CAROTID ARTERY | Peripheral endovascular |
| L312 | ARTERIOGRAPHY OF CAROTID ARTERY | Peripheral endovascular |
| L318 | TRANSLUMINAL OPERATIONS ON CAROTID ARTERY (O) | Peripheral endovascular |
| L319 | TRANSLUMINAL OPERATIONS ON CAROTID ARTERY (U) | Peripheral endovascular |
| L383 | OPEN EMBOLECTOMY OF SUBCLAVIAN ARTERY | Peripheral endovascular |
| L391 | PERCUTANEOUS TRANSLUMINAL ANGIOPLASTY OF SUBCLAVIAN ARTERY | Peripheral endovascular |
| L392 | PERCUTANEOUS TRANSLUMINAL EMBOLECTOMY OF SUBCLAVIAN ARTERY | Peripheral endovascular |
| L393 | PERCUTANEOUS TRANSLUMINAL EMBOLISATION OF SUBCLAVIAN ARTERY | Peripheral endovascular |
| L394 | ARTERIOGRAPHY OF SUBCLAVIAN ARTERY | Peripheral endovascular |
| L398 | TRANSLUMINAL OPERATIONS ON SUBCLAVIAN ARTERY (O) | Peripheral endovascular |
| L399 | TRANSLUMINAL OPERATIONS ON SUBCLAVIAN ARTERY (U) | Peripheral endovascular |
| L532 | OPEN EMBOLECTOMY OF ILIAC ARTERY | Peripheral endovascular |
| L541 | PERCUTANEOUS TRANSLUMINAL ANGIOPLASTY OF ILIAC ARTERY | Peripheral endovascular |
| L542 | PERCUTANEOUS TRANSLUMINAL EMBOLECTOMY OF ILIAC ARTERY | Peripheral endovascular |
| L543 | ARTERIOGRAPHY OF ILIAC ARTERY | Peripheral endovascular |
| L548 | TRANSLUMINAL OPERATIONS ON ILIAC ARTERY (O) | Peripheral endovascular |
| L549 | TRANSLUMINAL OPERATIONS ON ILIAC ARTERY (U) | Peripheral endovascular |
| L622 | OPEN EMBOLECTOMY OF FEMORAL ARTERY | Peripheral endovascular |
| L631 | PERCUTANEOUS TRANSLUMINAL ANGIOPLASTY OF FEMORAL ARTERY | Peripheral endovascular |
| L632 | PERCUTANEOUS TRANSLUMINAL EMBOLECTOMY OF FEMORAL ARTERY | Peripheral endovascular |
| L633 | PERCUTANEOUS TRANSLUMINAL EMBOLISATION OF FEMORAL ARTERY | Peripheral endovascular |
| L634 | ARTERIOGRAPHY OF FEMORAL ARTERY | Peripheral endovascular |
| L638 | TRANSLUMINAL OPERATIONS ON FEMORAL ARTERY (O) | Peripheral endovascular |
| L639 | TRANSLUMINAL OPERATIONS ON FEMORAL ARTERY (U) | Peripheral endovascular |
| L711 | PERCUTANEOUS TRANSLUMINAL ANGIOPLASTY OF ARTERY NEC | Peripheral endovascular |
| L712 | PERCUTANEOUS TRANSLUMINAL EMBOLECTOMY OF ARTERY NEC | Peripheral endovascular |
| L713 | PERCUTANEOUS TRANSLUMINAL EMBOLISATION OF ARTERY NEC | Peripheral endovascular |
| L714 | PERCUTANEOUS TRANSLUMINAL CANNULATION OF ARTERY NEC | Peripheral endovascular |
| L715 | PERCUTANEOUS TRANSLUMINLAL DILATION OF ARTERY NEC | Peripheral endovascular |
| L718 | THERAPEUTIC TRANSLUMINAL OPERATIONS ON OTHER ARTERY (O) | Peripheral endovascular |
| L719 | THERAPEUTIC TRANSLUMINAL OPERATIONS ON OTHER ARTERY (U) | Peripheral endovascular |
| L721 | ARTERIOGRAPHY NEC | Peripheral endovascular |
| L723 | PERCUTANEOUS TRANSLUMINAL ANGIOSCOPY NEC | Peripheral endovascular |
| L728 | DIAGNOSTIC TRANSLUMINAL OPERATIONS ON OTHER ARTERY (O) | Peripheral endovascular |
| L729 | DIAGNOSTIC TRANSLUMINAL OPERATIONS ON OTHER ARTERY (U) | Peripheral endovascular |
| L678 | EXCISION OF OTHER ARTERY (O) | Other |
| L679 | EXCISION OF OTHER ARTERY (U) | Other |
| L688 | REPAIR OF OTHER ARTERY (O) | Other |
| L689 | REPAIR OF OTHER ARTERY (U) | Other |
| L701 | OPEN EMBOLECTOMY OF ARTERY NEC | Other |
| L702 | OPEN EMBOLISATION OF ARTERY NEC | Other |
| L703 | LIGATION OF ARTERY NEC | Other |
| L704 | OPEN CANNULATION OF ARTERY NEC | Other |
| L708 | OTHER OPEN OPERATIONS ON OTHER ARTERY (O) | Other |
| L709 | OTHER OPEN OPERATIONS ON OTHER ARTERY (U) | Other |
| L752 | REPAIR OF ACQUIRED ARTERIOVENOUS FISTULA | Other |
| L758 | OTHER ARTERIOVENOUS OPERATIONS (O) | Other |
| L759 | OTHER ARTERIOVENOUS OPERATIONS (U) | Other |
| L974 | OPERATIONS ON ARTERY NEC | Other |
| L975 | OPERATIONS ON VEIN NEC | Other |
| L978 | OTHER OPERATIONS ON BLOOD VESSEL (O) | Other |
| L979 | OTHER OPERATIONS ON BLOOD VESSEL (U) | Other |
| L671 | BIOPSY OF ARTERY NEC | Other |
| L722 | MONITORING OF ARTERIAL PRESSURE | Other |
| L741 | INSERTION OF ARTERIOVENOUS PROSTHESIS | Other |
| L742 | CREATION OF ARTERIOVENOUS FISTULA NEC | Other |
| L743 | ATTENTION TO ARTERIOVENOUS SHUNT | Other |
| L748 | ARTERIOVENOUS SHUNT (O) | Other |
| L749 | ARTERIOVENOUS SHUNT (U) | Other |
| L751 | EXCISION OF CONGENITAL ARTERIOVENOUS MALFORMATION | Other |
| L753 | EMBOLISATION OF ARTERIOVENOUS ABNORMALITY | Other |
| L972 | PEROPERATIVE ANGIOPLASTY | Other |
| L973 | ISOLATED LIMB PERFUSION | Other |
