# Supplementary material for: A prospective investigation of depression and adverse outcomes in patients undergoing vascular surgical interventions: A retrospective cohort study using a large mental health database in South London
Source: Eur Psychiatry. 2021 Jan 18;64(1):e13. doi: 10.1192/j.eurpsy.2021.2 (PMC8057466; doi:10.1192/j.eurpsy.2021.2)
Supplement: Supplementary file 1 [file epasup.zip › S092493382100002Xsup002.docx]

**Supplementary table 2: Post-operative vascular surgery outcomes for patients with depression compared with those from the general population by type of vascular surgery with adjustments**

|  | ***Index vascular surgery hospitalisation*** | | | | ***Subsequent readmission*** | |
| --- | --- | --- | --- | --- | --- | --- |
| Type of vascular surgery/ Adjustments | *Odds ratio for emergency route of admission* | | *Incidence rate ratio for length of stay* | *Odds Ratio for mortality during index admission* | *Odds Ratio for emergency readmission within 30 days* | *Incidence Rate Ratio for length of stay of 30-day readmission* |
| Aortic/visceral endovascular (n= 933) | | | | | | |
| Univariate | 2.00 (1.03, 4.01), 0.04 | | 2.09 (1.88, 2.33), <0.001 | 0.97 (0.13, 7.38), 0.98 | 3.05 (1.28, 7.27), 0.01 | 2.43 (1.98, 2.98), <0.001 |
| Model 1 | 2.00 (1.01, 4.03), 0.04 | | 2.08 (1.86, 2.32), <0.001 | 0.99 (0.13, 7.56), 0.99 | 3.11 (1.29, 7.49), 0.01 | 2.61 (2.12, 3.20), <0.001 |
| Model 2 | 1.96 (1.02, 3.96), 0.04 | | 2.00 (1.79, 2.23), <0.001 | 0.92 (0.12, 7.10), 0.94 | 3.27 (1.34, 7.97), 0.01 | 2.67 (2.17, 3.28), <0.001 |
| Model 3 | 1.88 (0.87, 4.07), 0.09 | | 1.83 (1.63, 2.06), <0.001 | 0.98 (0.11, 8.69), 0.98 | 2.78 (1.08, 7.16), 0.03 | 2.41 (1.81, 3.20), <0.001 |
| Model 4 | 1.36 (0.89, 2.84), 0.74 | | 1.32 (1.16, 1.49), <0.001 | 1.27 (0.14, 11.52), 0.83 | 2.45 (1.04, 6.93), 0.04 | 1.51 (1.12, 2.29), 0.03 |
| Model 5 |  | | 1.33 (1.18, 1.50), <0.001 | 0.94 (0.10, 8.54), 0.95 | 2.42 (1.02, 6.72), 0.05 | 1.34 (1.02, 2.03), 0.04 |
| Major open vascular (n=1,420) | | | | | | |
| Univariate | 1.19 (0.70, 2.01), 0.52 | | 1.59 (1.49, 1.70), <0.001 | 1.73 (0.60, 4.94), 0.31 | 1.61 (0.77, 3.34), 0.20 | 1.16 (0.94, 1.43), 0.16 |
| Model 1 | 1.16 (0.68, 1.96), 0.58 | | 1.63 (1.53, 1.74), <0.001 | 1.82 (0.63, 5.21), 0.27 | 1.65 (0.79, 3.44), 0.18 | 0.84 (0.67, 1.04), 0.10 |
| Model 2 | 1.26 (0.74, 2.15), 0.40 | | 1.65 (1.54, 1.76), <0.001 | 1.82 (0.63, 5.23), 0.27 | 1.38 (0.64, 3.00), 0.41 | 0.98 (0.79, 1.22), 0.88 |
| Model 3 | 1.15 (0.66, 2.01), 0.61 | | 1.46 (1.36, 1.56), <0.001 | 1.13 (0.37, 3.46), 0.84 | 1.10 (0.50, 2.43), 0.82 | 1.06 (0.85, 1.33), 0.60 |
| Model 4 | 1.04 (0.62, 1.78), 0.62 | | 1.20 (1.12, 1.29), <0.001 | 0.84 (0.24, 2.96), 0.79 | 1.11 (0.48, 2.53), 0.81 | 0.79 (0.62, 1.02), 0.07 |
| Model 5 |  | | 1.24 (1.15, 1.33), <0.001 | 0.89 (0.25, 3.09), 0.85 | 1.10 (0.48, 2.51), 0.82 | 0.79 (0.61, 1.02), 0.07 |
| Peripheral endovascular (n=3,984) | | | | | | |
| Univariate | 2.05 (1.55, 2.71), <0.001 | | 2.39 (2.30, 2.48), <0.001 | 1.28 (0.80, 2.04), 0.30 | 2.90 (2.01, 4.20), <0.001 | 0.93 (0.85, 1.03), 0.19 |
| Model 1 | 2.29 (1.71, 3.06), <0.001 | | 2.44 (2.35, 2.53), <0.001 | 1.35 (0.84, 2.17), 0.21 | 3.03 (2.09, 4.40), <0.001 | 1.13 (1.03, 1.25), 0.01 |
| Model 2 | 2.13 (1.59, 2.86), <0.001 | | 2.64 (2.46, 2.83), <0.001 | 1.31 (0.81, 2.10), 0.27 | 2.96 (2.03, 4.32), <0.001 | 1.14 (1.03, 1.26), 0.01 |
| Model 3 | 1.58 (1.16, 2.18), 0.01 | | 2.07 (1.99, 2.16), <0.001 | 0.97 (0.58, 1.65), 0.92 | 2.53 (1.71, 3.74), <0.001 | 0.96 (0.86, 1.07), 0.42 |
| Model 4 | 1.32 (1.01, 1.87), 0.05 | | 1.37 (1.31, 1.44), <0.001 | 0.54 (0.30, 0.99), 0.05 | 2.28 (1.48, 3.50), <0.001 | 0.76 (0.66, 0.87), <0.001 |
| Model 5 |  | | 1.47 (1.41, 1.53), <0.001 | 0.66 (0.37, 1.18), 0.16 | 2.32 (1.52, 3.55), <0.001 | 0.77 (0.67, 0.88), <0.001 |
| Other (n=2,925) | |  | | |  | |
| Univariate | 1.60 (1.12, 2.28), 0.01 | | 2.64 (2.46, 2.82), <0.001 | 2.35 (0.82, 6.71), 0.11 | 1.81 (1.08, 3.02), 0.02 | 0.73 (0.59, 0.90), <0.001 |
| Model 1 | 1.49 (1.04, 2.13), 0.03 | | 2.72 (2.54, 2.92), <0.001 | 2.70 (0.93, 7.79), 0.07 | 2.02 (1.20, 3.41), 0.01 | 0.83 (0.67, 1.03), 0.09 |
| Model 2 | 1.34 (0.93, 1.94), 0.12 | | 2.44 (2.34, 2.53), <0.001 | 2.52 (0.86, 7.34), 0.09 | 2.10 (1.24, 3.57), 0.01 | 0.81 (0.65, 1.01), 0.06 |
| Model 3 | 1.18 (0.80, 1.74), 0.42 | | 1.44 (1.33, 1.56), <0.001 | 1.39 (0.39, 4.95), 0.61 | 1.68 (0.95, 2.95), 0.07 | 0.90 (0.72, 1.12), 0.35 |
| Model 4 | 0.75 (0.49, 1.16), 0.20 | | 0.90 (0.82, 0.99), 0.03 | 1.19 (0.30, 4.72), 0.81 | 1.45 (0.78, 2.67), 0.24 | 1.06 (0.83, 1.36), 0.64 |
| Model 5 |  | | 1.10 (1.01, 1.20), 0.04 | 1.51 (0.39, 5.81), 0.55 | 1.47 (0.80, 2.70), 0.22 | 0.95 (0.74, 1.22), 0.69 |

Univariate is the unadjusted model; Model 1 (Adjusted for age and gender); Model 2 (Model 1+ ethnicity and IMD score); Model 3 (Model 2+ CVD hospital admission); Model 4 (Model 3+ physical disability related hospitalisation); Model 5 (Model 4+ emergency vascular hospital admission)
