# Supplementary material for: A prospective investigation of depression and adverse outcomes in patients undergoing vascular surgical interventions: A retrospective cohort study using a large mental health database in South London
Source: Eur Psychiatry. 2021 Jan 18;64(1):e13. doi: 10.1192/j.eurpsy.2021.2 (PMC8057466; doi:10.1192/j.eurpsy.2021.2)
Supplement: Supplementary file 1 [file epasup.zip › S092493382100002Xsup003.docx]

**Supplementary table 3: Univariate and multivariate analysis showing predictors of health outcomes during index vascular surgery spell for patients with depression; OR/ IRR (95% CI), P value**

|  | **Odds ratio for emergency route of admission*** | | | **Outcome: Length of stay (days) for the index hospitalisation^$^** | | | | **Outcome: Inpatient mortality during the index hospitalisation IRR (95% CI), P value^&^** | | |
| --- | --- | --- | --- | --- | --- | --- | --- | --- | --- | --- |
| **Predictors** | **Univariate**  **(n= 446)** | **Model 1**  **(n= 265)** | **Model 2**  **(n= 265)** | **Univariate**  **(n= 446)** | **Model 1**  **(n= 256)** | **Model 2**  **(n= 256)** | **Model 3**  **(n= 256)** | **Univariate**  **(n= 446)** | **Model 1**  **(n= 442)** | **Model 2**  **(n= 443)** |
| Index admission- admitted to hospital via Emergency route |  |  |  | 1.92 (1.58, 2.32) | 5.12 (4.62, 5.68) | 5.10 (4.61, 5.64) | 5.09 (4.61, 5.63) | 18.28 (4.29, 77.91) | 15.15 (3.46, 65.45) | 15.15 (3.48, 65.95) |
| **Sociodemographic characteristics** | | | | | | | | | | |
| 10 year increase in age at hospital admission | 1.14 (0.99, 1.26) | 0.97 (0.82, 1.15) |  | 1.07 (0.99, 1.13) | 1.02 (0.92, 1.14) |  |  | 1.16 (0.93, 1.44) |  |  |
| Male | 0.97 (0.67, 1.42) |  |  | 1.07 (1.01, 1.13) | 1.04 (0.96, 1.12) |  |  | 0.47 (0.21, 1.07) | 0.35 (0.14, 0.88) | 0.36 (0.15, 0.88) |
| Non-white ethnicity | 0.55 (0.35, 0.87) | 0.50 (0.29, 0.87) | 0.51 (0.30, 0.86) | 0.65 (0.61, 0.70) | 0.81 (0.74, 0.89) | 0.80 (0.73, 0.87) | 0.80 (0.73, 0.88) | 0.56 (0.21, 1.46) |  |  |
| 10-unit increase in IMD | 0.96 (0.82, 1.13) |  |  | 0.97 (0.94, 0.99) | 1.05 (1.01, 1.09) | 1.05 (1.01, 1.09) | 1.05 (1.01, 1.09) | 0.77 (0.56, 1.02) | 0.81 (0.57, 1.19) |  |
| **HoNoS problems** |  |  |  |  |  |  |  |  |  |  |
| Agitation problems | 0.92 (0.45, 1.87) |  |  | 0.89 (0.79, 0.99) | 1.22 (1.06, 1.42) | 1.16 (1.01, 1.34) | 1.15 (0.94, 1.37) |  |  |  |
| Self-injury problems | 1.44 (0.76, 2.74) |  |  | 0.95 (0.86, 1.05) |  |  |  | 1.26 (0.35, 4.52) |  |  |
| Substance abuse problems | 1.51 (0.72, 3.20) |  |  | 0.72 (0.63, 0.81) | 0.80 (0.68, 0.95) | 0.83 (0.71, 0.97) | 0.84 (0.72, 0.99) | 0.53 (0.07, 4.14) |  |  |
| Cognitive problems | 1.86 (1.05, 3.29) | 2.08 (1.06, 4.08) | 1.99 (1.04, 3.80) | 1.12 (1.04, 1.21) | 1.39 (1.26, 1.55) | 1.44 (1.31, 1.6) | 1.47 (1.33, 1.62) | 0.92 (0.26, 3.27) |  |  |
| Physical health problems | 0.91 (0.57, 1.47) |  |  | 1.56 (1.44, 1.69) | 1.00 (0.89, 1.12) |  |  | 0.50 (0.19, 1.30) |  |  |
| Hallucinations | 0.95 (0.39, 2.29) |  |  | 0.58 (0.50, 0.69) | 0.49 (0.41, 0.61) | 0.50 (0.4, 0.62) | 0.50 (0.40, 0.61) |  |  |  |
| Depressed | 1.01 (0.66, 1.56) |  |  | 1.25 (1.17, 1.32) | 1.00 (0.91, 1.08) |  |  | 0.77 (0.29, 1.99) |  |  |
| Relationship problems | 1.15 (0.70, 1.89) |  |  | 1.06 (0.99, 1.14) |  |  |  | 0.78 (0.25, 2.45) |  |  |
| Daily living problems | 1.27 (0.82, 1.97) |  |  | 1.55 (1.46, 1.65) | 1.45 (1.32, 1.59) | 1.45 (1.33, 1.58) | 1.45 (1.34, 1.58) | 0.54 (0.19, 1.58) |  |  |
| Living conditions problems | 1.30 (0.72, 2.33) |  |  | 1.12 (1.03, 1.21) | 0.83 (0.73, 0.93) | 0.83 (0.74, 0.93) | 0.83 (0.74, 0.93) | 1.09 (0.30, 3.92) |  |  |
| Occupational problems | 1.02 (0.63, 1.64) |  |  | 0.97 (0.90, 1.03) |  |  |  | 0.46 (0.13, 1.64) |  |  |
| **Psychiatric diagnosis** | | | | | | | | | | |
| Mental and behavioural disorders due to psychoactive substance use (F10- F19) | 3.80 (1.48, 9.76) | 1.71 (0.57, 5.15) |  | 1.09 (0.97, 1.22) |  |  |  | 1.33 (0.30, 5.96) |  |  |
| Dementia | 3.06 (0.94, 9.91) |  |  | 1.16 (1.02, 1.32) | 0.64 (0.54, 0.75) | 0.64 (0.55, 0.76) | 0.67 (0.57, 0.78) | 2.50 (0.53, 11.74) |  |  |
| Neurotic, stress-related and somatoform disorders (F40- F48) | 2.38 (0.98, 5.24) | 1.82 (0.59, 5.60) |  | 1.57 (1.45, 1.71) | 1.30 (1.12, 1.49) | 1.31 (1.15, 1.50) | 1.33 (1.16, 1.52) | 0.50 (0.07, 3.78) |  |  |
| Disorders of adult personality and behaviour (F60- F69) | 1.82 (0.73, 4.55) |  |  | 0.83 (0.73, 0.96) | 0.80 (0.63, 1.10) |  |  | 0.75 (0.1, 5.79) |  |  |
| **Psychiatric medication before vascular surgery** | | | | | | | | | | |
| Antipsychotics | 1.40 (0.84, 2.34) |  |  | 1.19 (1.11, 1.28) | 1.19 (1.06, 1.34) | 1.14 (1.02, 1.28) | 1.15 (1.02, 1.29) | 0.85 (0.29, 2.53) |  |  |
| Antidepressants | 0.96 (0.66, 1.40) |  |  | 1.18 (1.12, 1.25) | 1.11 (1.01, 1.22) | 1.11 (1.01, 1.23) | 1.11 (1.01, 1.22) | 0.54 (0.24, 1.21) |  |  |
| Anxiolytics and Hypnotics | 1.07 (0.67, 1.71) |  |  | 1.07 (1.01, 1.14) | 0.69 (0.61, 0.78) | 0.66 (0.59, 0.74) | 0.67 (0.59, 0.75) | 0.46 (0.14, 1.55) |  |  |
| **Physical health medication** | | | | | | | | | | |
| Anticoagulants | 2.48 (1.26, 4.87) | 1.81 (0.78, 4.22) |  | 1.24 (1.15, 1.35) | 0.97 (0.86, 1.09) |  |  | 0.34 (0.04, 2.54) |  |  |
| Antidiabetics | 1.66 (0.75, 3.71) |  |  | 1.86 (1.71, 2.03) | 0.85 (0.72, 1.01) |  |  |  |  |  |
| Analgesics | 0.92 (0.56, 1.51) |  |  | 1.35 (1.27, 1.44) | 1.25 (1.12, 1.41) | 1.19 (1.08, 1.32) | 1.21 (1.09, 1.33) | 0.34 (0.08, 1.45) |  |  |
| Antihypertensives | 0.74 (0.46, 1.18) |  |  | 1.08 (1.01, 1.16) | 0.84 (0.75, 0.94) | 0.80 (0.72, 0.89) | 0.78 (0.71, 0.86) | 0.45 (0.13, 1.52) |  |  |
| **Previous physical disability related admissions** | | | | | | | | | | |
| Syncope and Collapse | 1.49 (0.99, 2.21) | 1.63 (0.94, 2.84) |  | 1.51 (1.43, 1.59) | 0.95 (0.88, 1.13) |  | 0.88 (0.81, 0.95) | 1.18 (0.54, 2.57) |  |  |
| Osteoporosis | 1.64 (0.82, 3.30) |  |  | 1.12 (1.03, 1.23) | 0.76 (0.67, 0.87) | 0.73 (0.64, 0.82) | 0.72 (0.64, 0.82) | 0.86 (0.20, 3.79) |  |  |
| UTIs | 2.42 (1.49, 3.93) | 2.00 (1.03, 3.91) | 2.59 (1.39, 4.83) | 1.76 (1.67, 1.86) | 1.42 (1.29, 1.55) | 1.43 (1.30, 1.56) | 1.41 (1.29, 1.54) |  |  |  |
| **Previous CVD hospital admissions** | | | | | | | | | | |
| Arrhythmia | 2.03 (1.28, 3.23) | 1.55 (0.82, 2.95) |  | 1.28 (1.21, 1.36) | 0.92 (0.74, 1.10) |  |  | 4.44 (2.06, 9.58) | 5.28 (2.11, 13.22) | 5.29 (2.12, 13.21) |
| Ischaemia and Coronary Heart Disease | 0.86 (0.57, 1.29) |  |  | 1.36 (1.29, 1.44) | 1.7 (1.53, 1.88) | 1.68 (1.52, 1.86) | 1.65 (1.49, 1.82) | 0.48 (0.18, 1.27) |  |  |
|  |  |  |  |  |  |  |  |  |  |  |
| Hypertension | 0.92 (0.62, 1.34) |  |  | 1.20 (1.13, 1.27) | 0.88 (0.79, 0.98) | 0.91 (0.82, 1.00) |  | 0.51 (0.24, 1.02) | 0.31 (0.12, 0.78) | 0.29 (0.11, 0.71) |
| Hypotension | 1.42 (0.82, 2.45) |  |  | 1.68 (1.58, 1.79) | 1.42 (1.26, 1.59) | 1.41 (1.26, 1.58) | 1.42 (1.27, 1.58) | 2.67 (1.13, 6.35) | 2.35 (0.97, 6.35) | 2.45 (1.02, 6.58) |
| Diabetes | 0.98 (0.66, 1.45) |  |  | 1.45 (1.37, 1.53) | 1.20 (1.08, 1.32) | 1.12 (1.03, 1.21) | 1.15 (1.07, 1.25) | 0.69 (0.30, 1.58) |  |  |
| Heart Failure | 1.53 (0.89, 2.62) |  |  | 2.22 (2.09, 2.35) | 1.88 (1.71, 2.06) | 1.85 (1.69, 2.03) | 1.86 (1.70, 2.04) | 1.29 (0.47, 3.51) |  |  |
| Hypercholesterolemia | 0.82 (0.55, 1.23) |  |  | 1.09 (1.04, 1.16) | 0.68 (0.62, 0.76) | 0.70 (0.63, 0.77) | 0.69 (0.62, 0.76) | 0.54 (0.22, 1.37) |  |  |

^&^ Model 1: AIC=170.8 ; BIC=199.4 ; Model 2: AIC=170.2 ; BIC=194.8

^$^ Model 1: AIC= 3695.3; BIC=3815.8; Model 2: AIC=3702.6; BIC=3818.4; Model 3: AIC=3708.8; BIC=3819.5;

^*^ Model 1: AIC= 353.6; BIC= 367.9; Model 2: AIC= 354.4; BIC= 390.2
