# Supplementary material for: A prospective investigation of depression and adverse outcomes in patients undergoing vascular surgical interventions: A retrospective cohort study using a large mental health database in South London
Source: Eur Psychiatry. 2021 Jan 18;64(1):e13. doi: 10.1192/j.eurpsy.2021.2 (PMC8057466; doi:10.1192/j.eurpsy.2021.2)
Supplement: Supplementary file 1 [file epasup.zip › S092493382100002Xsup004.docx]

**Supplementary table 4: Univariate and multivariate analysis showing predictors of health outcomes during emergency readmission to hospital within 30 days after discharge from hospital after vascular surgery spell for patients with depression; OR/ IRR (95% CI), P value**

|  | **Outcome: odds of emergency hospital admission within 30 days after index discharge^$^** | | | **Outcome: Length of stay for emergency hospital admission within 30 days after index discharge ^&^** | | | |
| --- | --- | --- | --- | --- | --- | --- | --- |
| **Predictors** | **Univariate**  **(n= 446)** | **Model 1***  **(n= 441)** | **Model 2* (n= 443)** | **Univariate (n= 446)** | **Model 1* (n= 255)** | **Model 2* (n= 333)** | **Model 3* (n= 338)** |
| Index admission- admitted to hospital via Emergency route | 2.12 (1.26, 3.58) | 1.87 (1.07, 3.27) | 1.99 (1.15, 3.43) | 1.92 (1.58, 2.32) | 1.11 (0.7, 1.76) |  |  |
| **Sociodemographic characteristics** | | | | | | | |
| 10 year increase in age at hospital admission | 1.09 (0.91, 1.28) |  |  | 1.02 (0.89, 1.16) |  |  |  |
| Male | 1.51 (0.87, 2.51) |  |  | 0.77 (0.67, 0.89) | 1.95 (1.39, 2.72) | 1.70 (1.38, 2.09) | 1.7 (1.4, 2.08) |
| Non-white ethnicity | 0.64 (0.34, 1.19) |  |  | 0.62 (0.51, 0.75) | 1.28 (0.92, 1.79) |  |  |
| 10-unit increase in IMD | 1.03 (0.83, 1.29) |  |  | 1.00 (0.84, 1.16) |  |  |  |
| **HoNoS problems** |  |  |  |  |  |  |  |
| Agitation problems | 0.91 (0.36, 2.29) |  |  | 0.64 (0.43, 0.93) | 2.75 (1.31, 5.79) | 1.62 (1.01, 2.59) | 1.61 (1.01, 2.57) |
| Self-injury problems | 1.27 (0.59, 2.73) |  |  | 0.60 (0.46, 0.79) | 0.18 (0.11, 0.30) | 0.40 (0.31, 0.58) | 0.41 (0.30, 0.58) |
| Substance abuse problems | 1.54 (0.66, 3.62) |  |  | 0.75 (0.56, 1.00) | 1.48 (0.61, 3.58) |  |  |
| Cognitive problems | 0.65 (0.29, 1.45) |  |  | 1.76 (1.44, 2.14) | 1.45 (1.02, 2.06) | 1.02 (0.82, 1.27) |  |
| Physical health problems | 1.09 (0.59, 2.02) |  |  | 1.64 (1.35, 2.00) | 0.92 (0.63, 1.35) |  |  |
| Hallucinations | 0.45 (0.10, 1.98) |  |  | 0.81 (0.46, 1.45) |  |  |  |
| Depressed | 0.73 (0.42, 1.27) |  |  | 1.84 (1.55, 2.17) | 2.19 (1.59, 3.03) | 1.66 (1.34, 2.06) | 1.67 (1.35, 2.06) |
| Relationship problems | 1.09 (0.59, 2.04) |  |  | 1.01 (0.84, 1.21) |  |  |  |
| Daily living problems | 0.92 (0.52, 1.61) |  |  | 1.74 (1.48, 2.05) | 0.95 (0.68, 1.34) |  |  |
| Living conditions problems | 0.85 (0.39, 1.85) |  |  | 0.63 (0.48, 0.83) | 1.19 (0.70, 2.02) |  |  |
| Occupational problems | 1.00 (0.55, 1.85) |  |  | 2.06 (1.75, 2.43) | 2.41 (1.42, 4.08) | 2.3 (1.81, 2.95) | 2.30 (1.82, 2.95) |
| **Psychiatric diagnosis** |  |  |  |  |  |  |  |
| Mental and behavioural disorders due to psychoactive substance use (F10- F19) | 2.75 (1.13, 6.68) | 2.64 (0.99, 7.03) | 2.52 (1.01, 6.50) | 0.63 (0.47, 0.84) | 0.31 (0.11, 0.88) | 0.34 (0.23, 0.5) | 0.34 (0.23, 0.51) |
| Dementia | 0.38 (0.05, 2.99) |  |  | 1.09 (0.73, 1.63) |  |  |  |
| Neurotic, stress-related and somatoform disorders (F40- F48) | 1.07 (0.39, 2.90) |  |  | 0.87 (0.63, 1.19) |  |  |  |
| Disorders of adult personality and behaviour (F60- F69) | 1.29 (0.42, 3.99) |  |  | 0.17 (0.07, 0.41) | 0.05 (0.02, 0.15) | 0.12 (0.04, 0.31) | 0.12 (0.04, 0.31) |
| **Psychiatric medication before vascular surgery** | | | | | | | |
| Antipsychotics | 1.50 (0.79, 2.82) |  |  | 0.57 (0.46, 0.72) | 1.30 (0.78, 2.19) |  |  |
| Antidepressants | 1.53 (0.92, 2.53) |  |  | 1.30 (1.13, 1.50) | 3.66 (2.51, 5.35) | 1.98 (1.62, 2.41) | 1.98 (1.62, 2.41) |
| Anxiolytics and Hypnotics | 1.08 (0.58, 2.02) |  |  | 0.87 (0.71, 1.05) |  |  |  |
| **Physical health medication** | | | | | | | |
| Anticoagulants | 1.27 (0.56, 2.87) |  |  | 0.44 (0.31, 0.64) | 0.66 (0.35, 1.26) |  |  |
| Antidiabetics | 2.94 (1.26, 6.89) | 1.85 (0.7, 4.84) |  | 0.99 (0.77, 1.27) |  |  |  |
| Analgesics | 1.74 (0.96, 3.18) |  |  | 1.12 (0.94, 1.33) |  |  |  |
| Antihypertensives | 1.29 (0.71, 2.36) |  |  | 0.97 (0.81, 1.16) |  |  |  |
| **Previous physical disability related admissions** | | | | | | | |
| Syncope and Collapse | 1.99 (1.20, 3.31) | 1.66 (0.95, 2.89) | 1.74 (1.02, 2.98) | 0.69 (0.60, 0.80) | 0.32 (0.24, 0.42) | 0.62 (0.52, 0.74) | 0.62 (0.52, 0.74) |
| Osteoporosis | 1.58 (0.69, 3.63) |  |  | 1.43 (1.17, 1.76) | 1.06 (0.69, 1.63) |  |  |
| UTIs | 2.14 (1.22, 3.75) | 1.46 (0.78, 2.73) |  | 3.49 (3.03, 4.02) | 2.77 (1.74, 4.43) | 2.03 (1.66, 2.48) | 2.03 (1.67, 2.48) |
| **Previous CVD hospital admissions** | | | | | | | |
| Arrhythmia | 0.93 (0.50, 1.73) |  |  | 0.95 (0.81, 1.11) |  |  |  |
| Ischaemia and Coronary Heart Disease | 2.13 (1.27, 3.57) | 1.53 (0.81, 2.9) |  | 1.74 (1.51, 2.00) | 0.99 (0.64, 1.53) |  |  |
| Hypertension | 1.76 (0.97, 3.04) | 1.02 (0.51, 2.05) |  | 1.21 (1.04, 1.41) | 0.84 (0.53, 1.32) |  |  |
| Hypotension | 1.33 (0.67, 2.65) |  |  | 1.06 (0.89, 1.26) |  |  |  |
| Diabetes | 1.77 (1.07, 2.94) | 1.26 (0.68, 2.34) |  | 1.48 (1.29, 1.70) | 0.86 (0.63, 1.19) |  |  |
| Heart Failure | 1.40 (0.72, 2.73) |  |  | 0.54 (0.42, 0.68) | 0.40 (0.26, 0.64) | 0.68 (0.52, 0.89) | 0.68 (0.52, 0.89) |
| Hypercholesterolemia | 2.17 (1.30, 3.62) | 1.75 (1.03, 3.31) | 2.39 (1.39, 4.10) | 3.24 (2.78, 3.78) | 1.14 (0.74, 1.74) |  |  |

^$^ Model 1: AIC=370.9; BIC=411.8; Model 2: AIC= 368.8; BIC=412.3;

^&^ Model 1: AIC= 1303.8; BIC= 948.0; Model 2: AIC= 1305.9; BIC=1404.4; Model 3: AIC= 1305.1; BC=1404.0;
